# ARID3B: a Novel Regulator of the Kaposi's Sarcoma-Associated Herpesvirus Lytic Cycle

**DOI:** 10.1128/JVI.03262-15

**Published:** 2016-09-29

**Authors:** Jennifer J. Wood, James R. Boyne, Christina Paulus, Brian R. Jackson, Michael M. Nevels, Adrian Whitehouse, David J. Hughes

**Affiliations:** a School of Molecular and Cellular Biology, Faculty of Biological Sciences, University of Leeds, Leeds, United Kingdom; b The Astbury Centre for Structural Molecular Biology, Faculty of Biological Sciences, University of Leeds, Leeds, United Kingdom; c Centre for Skin Sciences, University of Bradford, Bradford, United Kingdom; d Biomedical Sciences Research Complex, University of St. Andrews, St. Andrews, United Kingdom; Northwestern University

## Abstract

Kaposi's sarcoma-associated herpesvirus (KSHV) is the causative agent of commonly fatal malignancies of immunocompromised individuals, including primary effusion lymphoma (PEL) and Kaposi's sarcoma (KS). A hallmark of all herpesviruses is their biphasic life cycle—viral latency and the productive lytic cycle—and it is well established that reactivation of the KSHV lytic cycle is associated with KS pathogenesis. Therefore, a thorough appreciation of the mechanisms that govern reactivation is required to better understand disease progression. The viral protein replication and transcription activator (RTA) is the KSHV lytic switch protein due to its ability to drive the expression of various lytic genes, leading to reactivation of the entire lytic cycle. While the mechanisms for activating lytic gene expression have received much attention, how RTA impacts cellular function is less well understood. To address this, we developed a cell line with doxycycline-inducible RTA expression and applied stable isotope labeling of amino acids in cell culture (SILAC)-based quantitative proteomics. Using this methodology, we have identified a novel cellular protein (AT-rich interacting domain containing 3B [ARID3B]) whose expression was enhanced by RTA and that relocalized to replication compartments upon lytic reactivation. We also show that small interfering RNA (siRNA) knockdown or overexpression of ARID3B led to an enhancement or inhibition of lytic reactivation, respectively. Furthermore, DNA affinity and chromatin immunoprecipitation assays demonstrated that ARID3B specifically interacts with A/T-rich elements in the KSHV origin of lytic replication (oriLyt), and this was dependent on lytic cycle reactivation. Therefore, we have identified a novel cellular protein whose expression is enhanced by KSHV RTA with the ability to inhibit KSHV reactivation.

**IMPORTANCE** Kaposi's sarcoma-associated herpesvirus (KSHV) is the causative agent of fatal malignancies of immunocompromised individuals, including Kaposi's sarcoma (KS). Herpesviruses are able to establish a latent infection, in which they escape immune detection by restricting viral gene expression. Importantly, however, reactivation of productive viral replication (the lytic cycle) is necessary for the pathogenesis of KS. Therefore, it is important that we comprehensively understand the mechanisms that govern lytic reactivation, to better understand disease progression. In this study, we have identified a novel cellular protein (AT-rich interacting domain protein 3B [ARID3B]) that we show is able to temper lytic reactivation. We showed that the master lytic switch protein, RTA, enhanced ARID3B levels, which then interacted with viral DNA in a lytic cycle-dependent manner. Therefore, we have added a new factor to the list of cellular proteins that regulate the KSHV lytic cycle, which has implications for our understanding of KSHV biology.

## INTRODUCTION

Infection of immunocompromised people with Kaposi's sarcoma-associated herpesvirus (KSHV) is frequently linked with fatal malignancies. It is well established as the causative agent of primary effusion lymphoma (PEL) and Kaposi's sarcoma (KS) and is often associated with multicentric Castleman's disease (MCD) (1, 2). As with all herpesviruses, KSHV infection is lifelong and has two distinct phases to its life cycle: latency and the lytic cycle. Latency is associated with a highly restrictive viral gene expression program involving the latency-associated nuclear antigen (LANA), viral FLICE inhibitory protein (vFLIP), viral cyclin, kaposin, and various virally encoded microRNAs (miRNAs), and together, these are required for maintenance of the KSHV genome *in vitro* (3) and tumorigenesis *in vivo* (2). However, reactivation from latency to the lytic cycle is indispensable for the pathogenesis of KS; indeed, active virus replication and increased viral loads are associated with poorer clinical outcomes (4–6), and there is clinical evidence that treatment of patients with ganciclovir (inhibitor of herpesvirus replication) significantly reduces the incidence of KS (7). Therefore, a comprehensive understanding of molecular mechanisms that govern KSHV reactivation is critical to our understanding of disease progression.

Expression of a single viral protein, replication and transcriptional activator (RTA), is both necessary and sufficient for reactivation of the KSHV lytic cycle (reviewed in reference 8). *In vitro*, RTA expression is stimulated by certain cellular cues such as plasma cell differentiation (9, 10) and hypoxia (11), and because RTA autoactivates its own promoter and drives the full lytic cycle, it is well established as the true KSHV lytic switch protein (8). RTA activates transcription of lytic genes by directly interacting with RTA-responsive elements (RREs) found in some lytic gene promoters, or indirectly via interactions with cellular transcription factors, particularly RBP-Jκ, AP-1, and Oct-1 (8). The RTA protein comprises all the elements that one would expect for a transcriptional activator, such as nuclear localization signals (NLSs), a DNA binding domain (DBD), and a transcriptional activation (TA) domain. Further functional domains have also been identified, such as a leucine zipper domain that is important for homotetramer formation (12), a serine/threonine-rich region that is subject to phosphorylation (13), and a multitude of binding sites for various protein-protein interactions (PPIs) (8).

The best-characterized lytic promoters that are dependent on the direct DNA binding mechanism are the PAN and K12 promoters (14, 15). Both genes are highly expressed during lytic reactivation, but their expression is prevented when DNA binding mutants of RTA are expressed (15). Furthermore, transfection experiments with plasmids containing the PAN promoter showed that RTA was essential for activating transcription, as mRNA was undetectable in its absence (16). The sequence elements that were found to be RTA responsive demonstrated significant homology between the PAN and K12 promoters and centered on an A/T-rich trinucleotide (17) reminiscent of interferon-stimulated response elements found in the promoters of interferon-dependent genes (18).

There are several lytic genes that are activated via the indirect mechanism, including the RTA gene itself (*ORF50*), *ORF57* (*Mta*), *ORF6*, *ORF74* (*vGCPR*), *K6* (*vMIP-1*), and *ORF73* (*LANA*) (15, 19, 20). However, whether RTA's intrinsic DNA binding property is important for the activation of promoters via the indirect mechanism is a contentious issue. While some believe that RTA's ability to transactivate the ORF57 promoter is completely dependent on PPIs (15), others suggest that cooperation between RTA-DNA interactions and PPIs enhances the activation of transcription. For example, in addition to the essential RBP-Jκ binding site found in the ORF57 promoter, RTA binding sites have been identified surrounding this region and have been characterized as partial palindromes of A/T-rich elements with a 5′-CANT-3′ (N = any nucleotide) repeat core element (21). Extensive analysis of this promoter element suggested a model whereby RTA recruits RBP-Jκ to its consensus binding sequence, and through tetramer formation, RTA makes contacts with its cognate CANT repeats (21). Furthermore, stabilization of this complex may occur via further upstream CANT repeat interactions involving additional cellular transcription factors, such as AP-1 (8).

RTA is also required to coordinate lytic replication of the KSHV genome at the *cis*-acting origins of lytic replication (oriLyt). For example, it is responsible for docking the viral prereplication complex at the oriLyt (22), and this process, for unknown reasons, requires RTA-mediated transcription of an oriLyt-associated transcript (23, 24). Two distinct oriLyt sequences have been identified in the KSHV genome (oriLyt left and right); interestingly, RREs in oriLyt left share significant homology to those found in the PAN and K12 promoters (23). Furthermore, oriLyt contains an A/T-rich region consisting of various palindromic sequences in addition to a highly G/C-rich repeat element. Interestingly, DNA affinity studies have demonstrated that several cellular proteins preferentially interact with oriLyt via its A/T-rich regions (25).

Unlike most other herpesviruses, *de novo* infection of cell lines with KSHV leads to latency where the viral genome is rapidly chromatinized and lytic genes are associated with repressive histone modifications (26–28), making KSHV an amenable model for studying the establishment of herpesvirus latency. Furthermore, reactivation of the lytic cycle promotes various negative-feedback mechanisms that serve to temper lytic gene expression during reactivation, and *in vivo*, these presumably function to promote the establishment or maintenance of latency. These include viral factors (e.g., LANA [29, 30] and viral miRNAs [31]) and cellular proteins [e.g., NF-κB (32), TLE2 (33), poly(ADP-ribose) polymerase (PARP-1) (34), and histone deacetylase 1 (HDAC1) (35)] which have been found to temper RTA transactivation of its target promoters and thus reactivation of the lytic cycle. Clearly, silencing of KSHV lytic gene expression, or inhibition of lytic DNA replication, is an important step in the life cycle of KSHV. Nevertheless, the mechanisms that are involved are poorly understood.

To begin to gain an understanding of how RTA expression impacts the cellular proteome, we employed a global quantitative proteomics approach using stable isotope labeling of amino acids in cell culture coupled to tandem mass spectrometry (SILAC-MS/MS). Quantitative proteomics, using various platforms, is a powerful method that has been employed by various laboratories, including our own (36–38), to investigate virus-host interactions (recently reviewed in references 39 and 40). Using this approach, we identified a novel protein, A/T-rich interacting domain 3B (ARID3B), whose expression was enhanced by RTA and that regulated the KSHV lytic cycle. ARID3B, so called as it contains an A/T-rich interactive domain (ARID), belongs to a family of proteins involved in the regulation of gene expression. The ARID of ARID3A (a paralogue of ARID3B that functions as a B cell activator) has been shown to preferentially bind AATTAA sequences (41, 42); as this domain shares 89.9% sequence identity with that found in ARID3B, it suggests that there is functional conservation between these proteins.

As a transcription factor (43, 44), ARID3B promotes survival during development (44–46), and it is these properties that link it with various malignancies (43, 47–52). Very little else is known about ARID3B; however, we reasoned that its prosurvival properties may be important during the KSHV lytic cycle and it might promote lytic gene expression, thus explaining its RTA-mediated enhanced expression. However, we found that ARID3B inhibited lytic reactivation, and as it preferentially bound to regions containing A/T-rich elements in oriLyt, we consider the possibility that either it prevents oriLyt-dependent gene expression or it blocks access to proteins essential for viral genome replication.

## MATERIALS AND METHODS

### Cell lines, plasmids, and transfections.

TREx-BCBL-1-RTA cells (a kind gift from Jae Jung, University of Southern California) are a BCBL-1-based cell line that has been engineered to inducibly express exogenous Myc-tagged RTA by the addition of 1 μg/ml doxycycline hyclate, leading to a robust reactivation of the full KSHV lytic cycle (53); these were cultured in RPMI 1640 (Lonza) supplemented with 10% fetal bovine serum (FBS) (Life Technologies). Inducible SLK-BAC16 cells (iSLK-BAC16; also a gift from Jae Jung) were grown in Dulbecco modified Eagle medium (DMEM) (Lonza) supplemented with 10% FBS (Life Technologies). These maintain a latent infection with bacterial artificial chromosome 16 (BAC16)-derived KSHV (54). The parental SLK cell line, originally considered KSHV-negative endothelial cells derived from a KS patient, was subsequently found to be a contaminant of a renal carcinoma cell line, Caki1 (55). Nevertheless, these cells are widely used as a model for the study of KSHV biology. Initially, these cells were engineered to inducibly express RTA (iSLK) following the addition of doxycycline (56) and subsequently transfected with BAC16 and selected based on BAC-derived puromycin resistance (54). This cell line was demonstrated to support an authentic latent infection, and induction of the lytic cycle (with 1 μg/ml doxycycline hyclate) leads to the release of infectious virus (54).

HEK293T cells were used for reinfection assays as previously described (57). HEK293 rKSHV.219 cells (58) maintain KSHV as a latent infection and were generated by infecting HEK293T cells with a recombinant KSHV that contains a constitutively active puromycin resistance and green fluorescent protein (GFP) gene and a red fluorescent protein (RFP) gene that is fused to an RTA-responsive lytic cycle (PAN) promoter (not utilized in this study). Both HEK293T-based cell lines were maintained in DMEM (Lonza) supplemented with 10% FBS (Life Technologies).

To generate cells with inducible N-terminal FLAG-His-tagged RTA expression (iRTA-293), RTA was amplified from pRTS-ORF50 (14) (forward primer, 5′-ATCTTAAGGCCACCATGGATTATAAAGATGACGATGACAAGCATCATCATCATCATCATGCGCAAGATGACAAGGGTAAG-3′; reverse primer, 5′-ATCTCGAGTCAGTCTCGGAAGTAATTACG-3′) and ligated into the AflI-XhoI sites of pcDNA5-FRT-TO (Life Technologies) to generate pcDNA5-FH-RTA. Functionality of this vector was demonstrated due to its ability to reactivate the KSHV lytic cycle following its transfection. Flp-In-293 cells (Life Technologies) were transfected along with pOG44 (Flp recombinase) and subsequently selected using hygromycin B according to the manufacturer's protocol (Life Technologies). Clonal populations were generated by limiting dilution under hygromycin B selection, and clones with tightly regulated expression and normal growth properties (compared to parental cells) were selected. RTA expression was induced following treatment with 1 μg/ml doxycycline hyclate for the indicated times. iRTA-293 cells were maintained in DMEM (Lonza) supplemented with 10% FBS (Life Technologies).

The vector expressing C-terminally FLAG-tagged ARID3B was generated by PCR amplification using an ARID3B-containing plasmid (kind gift from Karen Cowden Dahl, Indiana University) as the template (forward primer, 5′-CGCGGATCCAAGCGATGGAGCCACTTCAGCAGCAGCAGCA-3′; reverse primer, 5′-CGCGAATTCTCACTTATCGTCGTCATCCTTGTAATCGAGGGACCAGCTGGTGGAGGGCTC-3′). Products were digested and ligated into the BamHI and EcoRI sites of pcDNA3 (pcDNA3-ARID3B-FLAG). FLAG-tagged SRAG was a kind gift from Stuart Wilson (University of Sheffield). Expression constructs were verified by DNA sequencing.

For transfections, cells were plated into 6-well plates, and transfections routinely used 1 μg plasmid DNA and Lipofectamine 2000 (Life Technologies) according to the manufacturer's instructions.

To determine the effects of overexpressed proteins on virus reactivation efficiencies, iSLK-BAC16 cells were transfected for 24 h and the lytic cycle was induced by doxycycline treatment for a further 24 h. Samples were processed for immunoblot analysis (see below).

### Stable isotope labeling of amino acids in cell culture (SILAC) proteomics.

iRTA-293 cells were grown in “heavy”-labeled medium containing stable isotopes of arginine (R) and lysine (K) (R10K8; DMEM-16; Dundee Cell Products) or “light”-labeled medium (R0K0; DMEM-14; Dundee Cell Products) supplemented with 10% dialyzed fetal calf serum (FCS) (DS1003; Dundee Cell Products) for 2 weeks. Cells were grown to 80 to 90% confluence in 10-cm dishes that included poly-l-lysine-coated coverslips, used to verify RTA expression by immunofluorescence (see below). RTA expression was induced for 12 h following treatment of heavy-labeled cells with 1 μg/ml doxycycline hyclate. To reduce sample complexity and to investigate how RTA expression impacts the nuclear proteome, labeled cells were fractionated (verified by immunoblotting; see below). After the monolayer was rinsed with phosphate-buffered saline (PBS), 1 × 10^7^ cells from each culture were lysed in 5 ml cytoplasmic lysis buffer containing 20 mM Tris (pH 7.4), 100 mM NaCl, 0.5 mM EDTA, 0.5% NP-40, and 1× protease inhibitor cocktail (Roche) for 20 min at 4°C. The nuclei were pelleted at 2,000 × *g* for 5 min at 4°C, and the cytoplasmic fraction was removed and stored. Nuclei were washed three times in cytoplasmic lysis buffer, and then nuclear proteins were extracted using 200 μl radioimmunoprecipitation assay (RIPA) buffer by repeated pipetting and storage on ice for 15 min. Insoluble material was pelleted at 12,000 × *g* for 10 min, and the supernatant was removed.

Equal amounts of protein from unlabeled and labeled samples were combined prior to protein digestion. Briefly, samples were reduced in 50 mM dithiothreitol (DTT)-1× NuPage LDS loading buffer and then separated by one-dimensional SDS-PAGE (4 to 12% Bis-Tris Novex minigel; Life Technologies) and visualized by colloidal Coomassie blue staining (Novex; Life Technologies). The entire protein gel lanes were excised and cut into 10 slices each. Every gel slice was subjected to in-gel digestion with trypsin overnight at 37°C. The resulting tryptic peptides were extracted by formic acid (1%) and acetonitrile, lyophilized in a Speed Vac, and resuspended in 1% formic acid. Trypsin-digested peptides were separated using an Ultimate 3000 RSLC (Thermo Scientific) nanoflow liquid chromatography (LC) system. On average, 0.5 μg was loaded with a constant flow of 5 μl/min onto an Acclaim PepMap100 nanoViper C_18_ trap column (100-μm inner diameter, 2 cm; Thermo Scientific). After trap enrichment, peptides were eluted onto an Acclaim PepMap RSLC nanoViper C_18_ column (75 μm, 15 cm; Thermo Scientific) with a linear gradient of 2 to 40% solvent B (80% acetonitrile with 0.08% formic acid) over 65 min with a constant flow of 300 nl/min. The high-performance liquid chromatography (HPLC) system was coupled to a linear ion trap Orbitrap hybrid mass spectrometer (LTQ Orbitrap Velos; Thermo Scientific) via a nanoelectrospray ion source (Thermo Scientific). The spray voltage was set to 1.2 kV, and the temperature of the heated capillary was set to 250°C. Full-scan MS survey spectra (*m/z* 335 to 1,800) in profile mode were acquired in the Orbitrap with a resolution of 60,000 after accumulation of 1,000,000 ions. The 15 most intense peptide ions from the preview scan in the Orbitrap were fragmented by collision-induced dissociation (normalized collision energy, 35%; activation Q, 0.250; activation time, 10 ms) in the LTQ after the accumulation of 10,000 ions. Maximal filling times were 1,000 ms for the full scans and 150 ms for the MS/MS scans. Precursor ion charge state screening was enabled, and all unassigned charge states as well as singly charged species were rejected. The lock mass option was enabled for survey scans to improve mass accuracy. Data were acquired using the Xcalibur software. The raw mass spectrometric data files obtained for each experiment were collated into a single quantitated data set using MaxQuant (version 1.2.2.5) and the Andromeda search engine software. Enzyme specificity was set to that of trypsin, allowing for cleavage N terminal to proline residues and between aspartic acid and proline residues. Other parameters used were (i) variable modifications, methionine oxidation, protein N-acetylation, and Gln to pyro-Glu; (ii) fixed modifications, cysteine carbamidomethylation; (iii) database, target-decoy human MaxQuant (ipi.HUMAN.v3.68); (iv) heavy labels, R10K8; (v) MS/MS tolerance, Fourier transform mass spectroscopy (FTMS), 10 ppm; ion trap mass spectroscopy (ITMS), 0.6 Da; (vi) maximum peptide length, 6; (vii) maximum missed cleavages, 2; (viii) maximum of labeled amino acids, 3; and (ix) false discovery rate (FDR), 1%. Peptide ratios were calculated for each arginine- and/or lysine-containing peptide as the peak area of labeled arginine/lysine divided by the peak area of nonlabeled arginine/lysine for each single-scan mass spectrum. Peptide ratios for all arginine- and lysine-containing peptides sequenced for each protein were averaged. Data were normalized using 1/median ratio value for each identified protein group per labeled sample. Pathway analysis was performed using DAVID Bioinformatics Resources (https://david.ncifcrf.gov/) (59, 60) using proteins that were at least 2-fold different.

### Immunoprecipitation and immunoblotting.

Immunoprecipitation assays have been described previously (61). For immunoblot analysis, cells were washed in PBS and proteins were extracted in lysis buffer containing 50 mM Tris (pH 7.4), 150 mM NaCl, 1% NP-40, and 1× protease inhibitor cocktail (Roche) for 15 min on ice and clarified by centrifugation at 12,000 × *g* for 10 min, 4°C. For the detection of ARID3B by immunoblotting, we found that sonication prior to loading improved detection. SDS-PAGE and immunoblotting of normalized protein concentrations followed standard techniques using the following antibodies: rabbit polyclonal antibody (PAb) anti-FLAG tag (1:1,000; Sigma), mouse monoclonal antibody (MAb) anti-ORF57 207.6 (1:1,000; Santa Cruz), RTA, rabbit antiserum (1:400; gift from David Blackbourn, University of Surrey), mouse MAb anti-glyceraldehyde-3-phosphate dehydrogenase (anti-GAPDH) (1:5,000; Sigma), mouse MAb anti-lamin B1 (1:1,000; Santa Cruz), rabbit anti-ARID3B (1:200; Abcam), anti-ORF59 (Autogen Bioclear), and sheep anti-KSHV minor capsid protein (mCP; 1:1,000; Exalpha Biologicals, Inc.). Proteins were detected by chemiluminescence (using horseradish peroxidase [HRP]-conjugated secondary antibodies), and signals were captured digitally using the ChemiDoc MP Imager (Bio-Rad), which ensures that signal saturation does not occur. ImageLab software version 4.1 (Bio-Rad) was used to select and determine the background-subtracted density of the bands in all blots, and these were normalized against expression of GAPDH.

### Indirect immunofluorescence microscopy.

As previously described (62), coverslips were coated with poly-l-lysine and TREx-BCBL-1-RTA cells were plated (1 × 10^6^ per well of a 12-well plate) and doxycycline hyclate treated (1 μg/ml) for 18 h at 37°C. Following doxycycline hyclate treatment of SILAC-labeled iRTA-293 cells (see above), coverslips were removed from the 10-cm dishes and placed in a humidity chamber. Cells were gently washed with PBS, fixed using 4% formaldehyde (in PBS) for 10 min, permeabilized with PBS–1% Triton X-100 for 10 min, and washed three times with PBS as previously reported (62). Primary antibodies were diluted in PBS-2% bovine serum albumin (BSA), added to cells, and incubated in humidity chambers for 2 h at 37°C or overnight at 4°C followed by 5 washes with PBS. The appropriate secondary antibodies (Alexa Fluor 488 or 594; Life Technologies) were diluted 1:500 in PBS-2% BSA and incubated with cells for 1 h at 37°C followed by 5 washes with PBS. Coverslips were mounted in Vectashield with 4′,6-diamidino-2-phenylindole (DAPI) (Vector Laboratories).

Incorporation of ethyldeoxyuridine (EdU) was performed using a Click-iT EdU imaging kit (Life Technologies) (62). Briefly, 1 × 10^6^ TREx-BCBL-1-RTA cells were treated with doxycycline and the cells were plated onto poly-l-lysine-coated coverslips. After 16 h, cells were pulsed for 45 min with 10 μM EdU, washed, fixed, and permeabilized according to the manufacturer's recommendations. After detection of EdU (according to the manufacturer's protocol), the cells were washed and further incubated with the indicated antibody for 1 h at 37°C, washed again, and incubated with Alexa Fluor 488 goat anti-rabbit antibody (Life Technologies; 1:500 in PBS-2% BSA) for 1 h at 37°C. Finally, DNA was stained using Hoechst 33342 (1:20,00 in PBS) and mounted in Vectashield (Vector Laboratories). Images were captured using an LSM510 or LSM700 laser scanning microscope (Carl Zeiss) and processed using Zen imaging software (Carl Zeiss).

Antibodies included rabbit anti-FLAG (1:250; Sigma), mouse anti-Myc tag 9E10 (1:250; Sigma), RTA rabbit antiserum (1:100), and rabbit anti-ARID3B (1:100; Abcam).

### Quantitative PCR.

For reverse transcriptase-quantitative PCR (RT-qPCR), total cellular RNA was extracted from cells using TRIzol (Life Technologies) according to the manufacturer's instructions and contaminating DNA was removed using the DNA-free kit (Ambion). cDNA was generated from 1 μg RNA in 20-μl reaction volumes using Moloney murine leukemia virus (M-MuLV) reverse transcriptase (RT; New England BioLabs) according to the manufacturer's recommendations with 5 ng oligo(dT). In parallel, negative-control reactions were performed for each RNA by omitting RT in order to confirm that quantification represented cDNA and not contaminating DNA. For quantification of viral DNA, 1 × 10^6^ cells were treated with doxycycline hyclate or left untreated. At 72 h postreactivation, total DNA was extracted using a DNA minikit (Qiagen) and quantified by UV spectrophotometry. Viral DNA was quantified using primers specific for the ORF57 gene. Quantitative PCR mixtures (20 μl) included 1× SensiMix SYBR green master mix (Bioline), 0.5 μM (each) primer, and 1 μl cDNA or 3.4 ng total DNA. Cycling was performed in a RotorGene Q machine (Eppendorf) and included an initial 10-min denaturation step at 94°C, followed by 40 cycles of 30 s at 94°C, 30 s at 60°C, and 30 s at 72°C. Melting curve analysis was performed between 65 and 95°C (with 0.2°C increments) to verify amplicon specificity. Quantification of GAPDH mRNA (qRT-PCR) or GAPDH DNA (virus reactivation) was used to normalize between samples, and the average cycle threshold (*C_T_*) was determined from three independent samples from independent cultures. Calculations were made using the ΔΔ*C_T_* method.

### RNA interference (RNAi).

Conditions for small interfering RNA (siRNA) knockdown of ARID3B followed previously reported protocols (57). Briefly, 20 nM SMARTpool:On-TARGETplus ARID3B siRNA (L-012219-00-0005; Dharmacon), Hs_ARID3B_3 FlexiTube GeneSolution (GS10620; Qiagen), or scramble control siRNA (siRNA controls from the respective manufacturer were used as controls) was transfected into iSLK-BAC16 cells for 72 h. For experiments that required reactivation of the lytic cycle, doxycycline was added to cells for 24 h, as stated above.

### *In vitro* DNA affinity assay.

Assays were performed according to the method in reference 25, with minor modifications: HEK293 rKSHV.219 cells in 6-well plates were transfected with pcDNA3-ARID3B-FLAG, and the lytic cycle was induced for 24 h. oriLyt sequences 3F, 9F, and 11F were amplified from the KSHV genome using 5′-biotinylated primers (primer sequences already described [25]) and a control region of the KSHV genome (RTA gene body; forward primer, 5′-GTCTACCTTCCGAGGATTATGG-3′; reverse primer, 5′-GATTCTGGCATGAGACCGCTTC-3′). PCR products were incubated with Dynabeads M-280–streptavidin according to the manufacturer's recommendations (Life Technologies). Affinity purification followed the previously described methods (25).

### ChIP.

Cells were cross-linked with 1% formaldehyde (Thermo Scientific; catalog no. 28908) for 10 min at room temperature. Glycine was added to a final concentration of 125 mM to stop the cross-linking reaction, and the samples were incubated for another 5 min at room temperature. Cells were washed twice with ice-cold PBS and lysed in FastChIP buffer (50 mM Tris-HCl [pH 7.5], 150 mM NaCl, 5 mM EDTA, 0.5 mM DTT, 0.5% IGEPAL CA-630, 1.0% Triton X-100) containing protease inhibitors (Roche) as described previously (63, 64). Isolated nuclei were resuspended in SDS lysis buffer (50 mM Tris-HCl [pH 8.1], 10 mM EDTA, 1% SDS), and the samples were incubated on ice for 15 min. Nuclear lysates were sonicated for 10 min in a Diagenode Bioruptor Pico (30-s on-off intervals) and cleared by centrifugation for 30 min at 20,000 × *g* and 4°C. Sheared chromatin from 9 × 10^6^ cells (180 μl) was combined with 9 volumes of chromatin immunoprecipitation (ChIP) dilution buffer (16.7 mM Tris-HCl [pH 8.1], 167 mM NaCl, 1.2 mM EDTA, 1.1% Triton X-100, 0.01% SDS) and subjected to immunoprecipitation for 16 h at 4°C with gentle rotation using 13 μl of rabbit anti-ARID3B antibody (Bethyl Laboratories; catalog no. A302-564A-M) or 4 μg of normal rabbit IgG. Samples were centrifuged for 10 min at 20,000 × *g*, 4°C, to remove any precipitated material, and the supernatants were combined with 20 μl Magna ChIP protein A magnetic beads (Millipore; catalog no. 16-661) and incubated for 2 h at 4°C with gentle rotation. Immune complexes were washed with 1 ml each of low-salt buffer (20 mM Tris-HCl [pH 8.1], 150 mM NaCl, 2 mM EDTA, 1% Triton X-100, 0.1% SDS), high-salt buffer (20 mM Tris [pH 8.1], 0.5 M NaCl, 2 mM EDTA, 1% Triton X-100, 0.1% SDS), and lithium chloride (LiCl) buffer (10 mM Tris [pH 8.1], 0.25 M LiCl, 1 mM EDTA, 1% IGEPAL CA-630, 1% deoxycholic acid), and twice with TE (10 mM Tris-HCl [pH 8.0], 1 mM EDTA) buffer. Elution of the chromatin-antibody complexes was carried out by incubation with 150 μl freshly prepared elution buffer (100 mM NaHCO_3_, 1% SDS) containing 1.5 μl proteinase K (Roche; catalog no. 03 115 887 001) at 62°C for 2 h, followed by a 10-min incubation step at 95°C. DNA was purified using the NucleoSpin gel and PCR cleanup kit from Macherey-Nagel (catalog no. 740609) according to their DNA cleanup protocol for samples containing SDS. DNA was eluted with 90 μl buffer NE, and 5 μl of the DNA solution was used as the template DNA for qPCR using primers specific for the A/T-rich region of oriLyt left (forward primer, 5′-CCCTCCTTTGTTTTCCGGAAG-3′; reverse primer, 5′CTCATCGGGCCCTATTATAAAG-3′) and the RTA coding region (forward primer, 5′-GTCTACCTTCCGAGGATTATGG-3′; reverse primer, 5′-GATTCTGGCATGAGACCGCTTC-3′).

## RESULTS

### ARID3B expression is enhanced by RTA.

**FIG 1:**
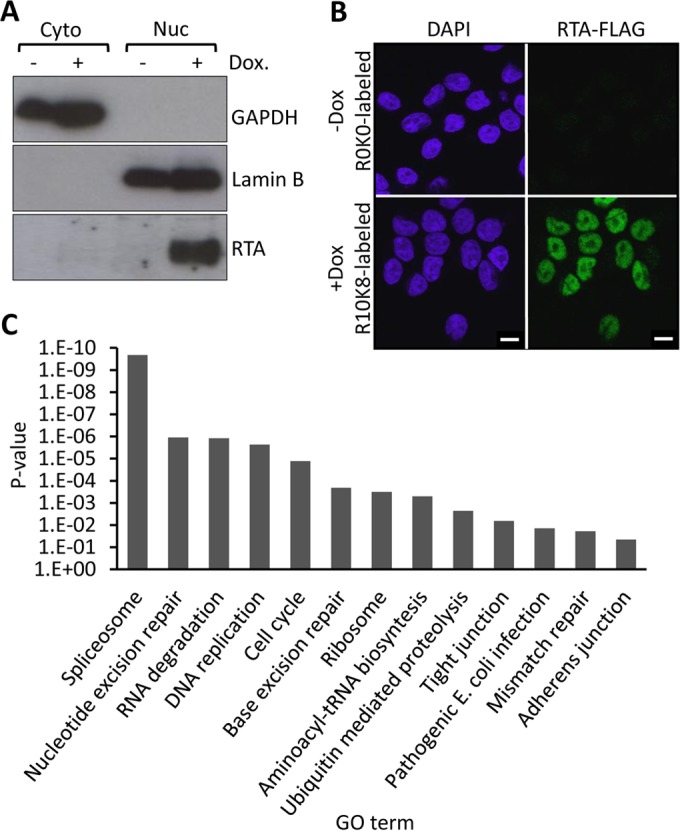
Analysis via SILAC coupled to LC-MS/MS of nuclear proteome changes in response to KSHV-RTA expression. Flp-In-293 cells were used to develop a cell line with doxycycline (Dox)-inducible FLAG-tagged RTA expression (iRTA-293). (A) Cells were labeled with R10K8 (heavy)- or R0K0 (light)-labeled SILAC medium for 2 weeks. FLAG-RTA expression was induced in heavy-labeled cells for 12 h, and these were fractionated into nuclear and cytoplasmic compartments. Fractionation success was validated by immunoblot analysis using the nuclear (Lamin-B1) and cytoplasmic (GAPDH) markers. As expected, RTA expression was largely restricted to the nuclear compartment and was found only in doxycycline-treated cells. (B) FLAG-RTA expression was analyzed by confocal immunofluorescence that revealed that all doxycycline-treated cells were positive for FLAG-RTA and untreated cells (-Dox; R0K0-labeled cells) were negative for RTA expression, demonstrating tight regulation of FLAG-RTA. Bars, 10 μm. (C) KEGG pathway analysis of nuclear proteins that were upregulated 2-fold or more due to RTA expression, with at least 2 independent peptides revealing various cellular pathways associated with RTA expression. GO term, gene ontology term.

To investigate the impact of the lytic switch protein RTA expression on the host proteome, we developed a cell line with doxycycline-inducible RTA expression (iRTA-293) and applied global proteomics using stable isotope labeling of amino acids in cell culture (SILAC). After metabolically labeling cells in media containing different stable isotopes of arginine (R) and lysine (K) (e.g., R10K8 [heavy] or R0K0 [light]) for 2 weeks, we induced RTA expression from heavy-labeled cells for 12 h while leaving light-labeled cells untreated. We tested RTA expression by immunofluorescence and then fractionated cells into cytoplasmic and nuclear fractions that were validated by immunoblot analysis using cytoplasmic and nuclear markers (Fig. 1A and B). Both these assays demonstrated the expected nuclear expression of RTA. We combined the nuclear fractions (heavy versus light) 1:1 and applied LC-MS/MS (Dundee Cell Products [see Materials and Methods]). Identified and quantified proteins with a minimum of two unique peptides and a change in abundance of >2-fold were taken forward for bioinformatics analyses. KEGG pathway analysis of the nuclear proteome of proteins increased in abundance upon RTA expression (using DAVID Bioinformatics Resources [59, 60]) identified various pathways that one might expect to be modulated upon RTA expression (e.g., cell cycle control [65] and DNA replication [24]) (Fig. 1C). However, various DNA damage response (DDR) pathways were also identified in addition to ubiquitin-mediated proteolysis (specifically Cullin-RING ubiquitin ligases [CRLs]). A cellular mechanism that regulates CRLs (66–68) and many of the listed DDR pathways (69–71) is the ubiquitin-like modification NEDDylation. Of note, we recently showed that blocking NEDDylation in cells latently infected with KSHV resulted in the activation of RTA expression but inhibited viral DNA replication (62). Interestingly, a similar observation (with regard to effects on viral gene expression and viral DNA replication) was recently reported for the betaherpesvirus human cytomegalovirus (HCMV); blocking of NEDDylation also led to increased immediate early gene expression in the absence of viral DNA replication (72). Furthermore, both of these reports showed that Cullin 4B was particularly important for regulating viral gene expression (62, 72).

In addition to investigating cellular pathways associated with RTA expression, we compiled a list of the top 15 proteins that showed increases in abundance in RTA-expressing cells (Table 1). Interestingly, a number of proteins associated with transcriptional repression were identified [cell division cycle-associated 7-like (15.5-fold increase), Polycomb group protein Lethal(3)malignant brain tumor-like protein 3 (5.7-fold increase), and lysine-specific demethylase 2A (5.2-fold increase)]. We also note that RTA expression was associated with an increased abundance of protein LYRIC (7.0-fold increase), which has been shown to activate NF-κB (Table 1); intriguingly, these data suggest that RTA is associated with the expression of factors that one would predict to inhibit RTA's transcriptional activity. In accordance with this, we also applied ingenuity pathway analysis (IPA) to the same data set, and the top canonical pathway identified, in addition to cell cycle control, DNA replication, and various DNA damage pathways, was “transcriptional repression” (Table 2). We are currently investigating some of these observations, and in this report, we focus on the novel protein ARID3B (7.5-fold increase). There are a number of features that led us to investigate ARID3B. Relatively little is known about this protein, and using KSHV as a tractable model system, we wished to further study its function. It has been shown that ARID3B functions as a transcription factor linked with prosurvival mechanisms during developmental processes (44–46), and these functions may be beneficial for reactivation. Furthermore, ARIDs have been described as DNA binding domains that favor A/T-rich sequences; given the predominance of A/T-rich elements in regulatory regions of the KSHV genome (RREs, oriLyt etc.), we hypothesized that ARID3B would cooperate with RTA during reactivation of the lytic cycle.

**TABLE 1:** Top 15 proteins increased in abundance in the nuclear fraction upon RTA expression

Name	UniProt identifier^*a*^	Fold increase	No. of unique peptides	Sequence coverage (%)	PEP^*b*^	Function^*a*^
ATPase family AAA domain-containing protein 3B	Q5T9A4-1	53.4	12	19.8	1.90E−79	May play a role in a mitochondrial network organization typical for stem cells
Cell division cycle-associated 7-like protein	Q96GN5-1	15.5	3	9.3	1.68E−18	Transcriptional repressor
Transmembrane protein 43	Q9BTV4	11.9	7	24.8	1.18E−55	May have a role in maintaining nuclear envelope structure
Kinesin-like protein KIF11	P52732	11.7	37	46.14	6.53E−283	Motor protein required for establishing a bipolar spindle during mitosis
BAT2 domain-containing protein 1	Q9Y520-7	10.8	39	17	1.39E−164	Unknown
Centrosomal protein of 170 kDa	Q5SW79-1	9.6	19	14.3	2.53E−66	Plays a role in microtubule organization
Uncharacterized protein KIAA1671	Q9BY89-1	9.3	4	2.6	3.61E−11	Unknown
Upstream of NRAS	Q68DF1	9.1	18	26.1	2.26E−193	May be involved in translationally coupled mRNA turnover
AT-rich interactive domain-containing protein 3B	Q8IVW6-1	7.5	8	15.7	2.25E−67	Transcription factor which may be involved in neuroblastoma growth and malignant transformation
General transcription factor 3C polypeptide 2	Q8WUA4-1	7.5	3	5.5	1.88E−10	Required for RNA polymerase III-mediated transcription
Protein LYRIC	Q86UE4	7.0	4	7.7	2.99E−14	Activates the NF-κB transcription factor
Cadherin-2	P19022	6.4	3	5.8	2.33E−11	Calcium-dependent cell adhesion proteins
Cytospin A	Q69YQ0	6.1	3	3.3	3.88E−10	Involved in cytokinesis and spindle organization
Lethal(3)malignant brain tumor-like 3 protein	Q96JM7-1	5.7	3	4	3.46E−07	Putative Polycomb group (PcG) protein; PcG proteins maintain the transcriptionally repressive state of genes
Lysine-specific demethylase 2A	Q9Y2K7-1	5.2	3	2.8	8.35E−08	Histone demethylase that specifically demethylates Lys-36 of histone H3; maintains heterochromatin

a Protein information was annotated from the UniProt database (www.uniprot.org).

b PEP, posterior error probability (a statistical measure for peptide identification—should be below 0.1).

**TABLE 2:** Ingenuity pathway analysis of the top canonical pathways associated with RTA expression (nuclear proteome)

Name	*P* value
Transcriptional repression	2.85E−8
Aminoacyl-tRNA biosynthesis	3.87E−5
Cell cycle control of chromosomal replication	7.64E−4
Mismatch repair in eukaryotes	7.84E−4
Role of BRCA1 in DNA damage response	8.12E−4

**FIG 2:**
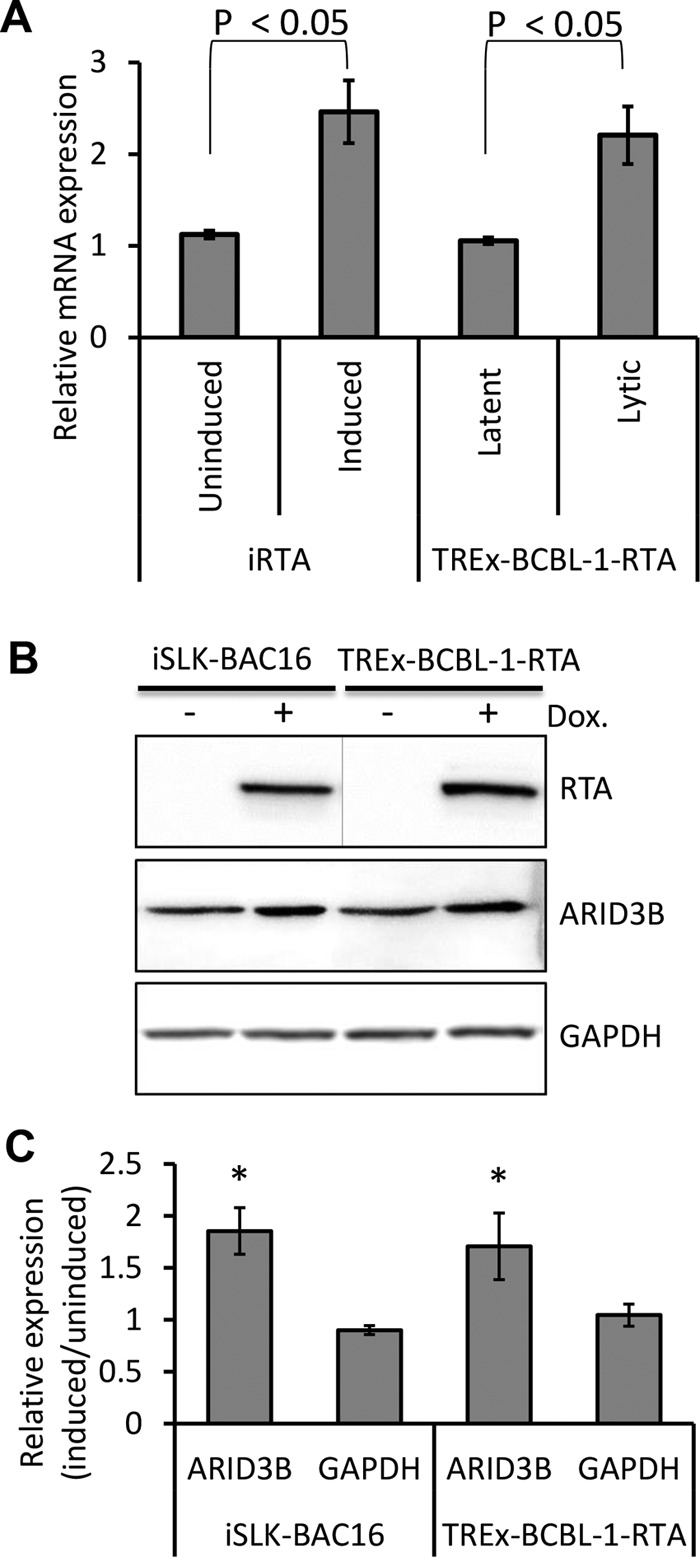
RTA enhances ARID3B expression. (A) Doxycycline-induced expression in iRTA-293 cells for 12 h and reactivation of the KSHV lytic cycle by the addition of doxycycline in TREx-BCBL1-RTA cells increased *ARID3B* expression as measured by qRT-PCR. Error bars represent standard deviations from the mean from three independent biological replicates, and quantification was normalized to *GAPDH* using the ΔΔ*C_T_* method. Student's *t* test was used to determine statistical significance. Three independent experiments were performed for each cell line. (B) Reactivation of the lytic cycle from latently infected iSLK-BAC16 and TREx-BCBL1-RTA cells led to an increase in ARID3B protein expression. In this experiment, separate immunoblots were used for the detection of RTA (denoted by a line). Dox., doxycycline. (C) Quantification of protein expression in panel B (see Materials and Methods). Data are derived from two independent experiments, and error bars represent standard deviations of the mean (calculated from technical replicates within independent experiments). *, *P* < 0.05 (Student's *t* test).

To investigate how RTA expression might lead to an increase in ARID3B levels, we induced RTA expression in iRTA-293 cells in addition to reactivating the lytic cycle in TREx-BCBL-1-RTA cells (see Materials and Methods for a description of the cell lines used in this study). We performed RT-qPCR analysis and found that RTA, and lytic reactivation, led to a small but reproducible increase in *ARID3B* mRNA expression (ca. 2.5-fold increase [Fig. 2A]). We also reactivated the lytic cycle in iSLK-BAC16 and TREx-BCBL-1-RTA cells and performed immunoblot analyses using antibodies specific for ARID3B (Fig. 2B). This showed that lytic reactivation led to an approximately 1.5- to 2-fold increase in ARID3B expression (Fig. 2C). These changes are modest but likely reflect the superior sensitivity of mass spectrometry and the semiquantitative nature of immunoblot data. Together, these data demonstrate that RTA, and the KSHV lytic cycle, enhances ARID3B expression at both the RNA and protein levels.

### ARID3B is relocalized during reactivation.

**FIG 3:**
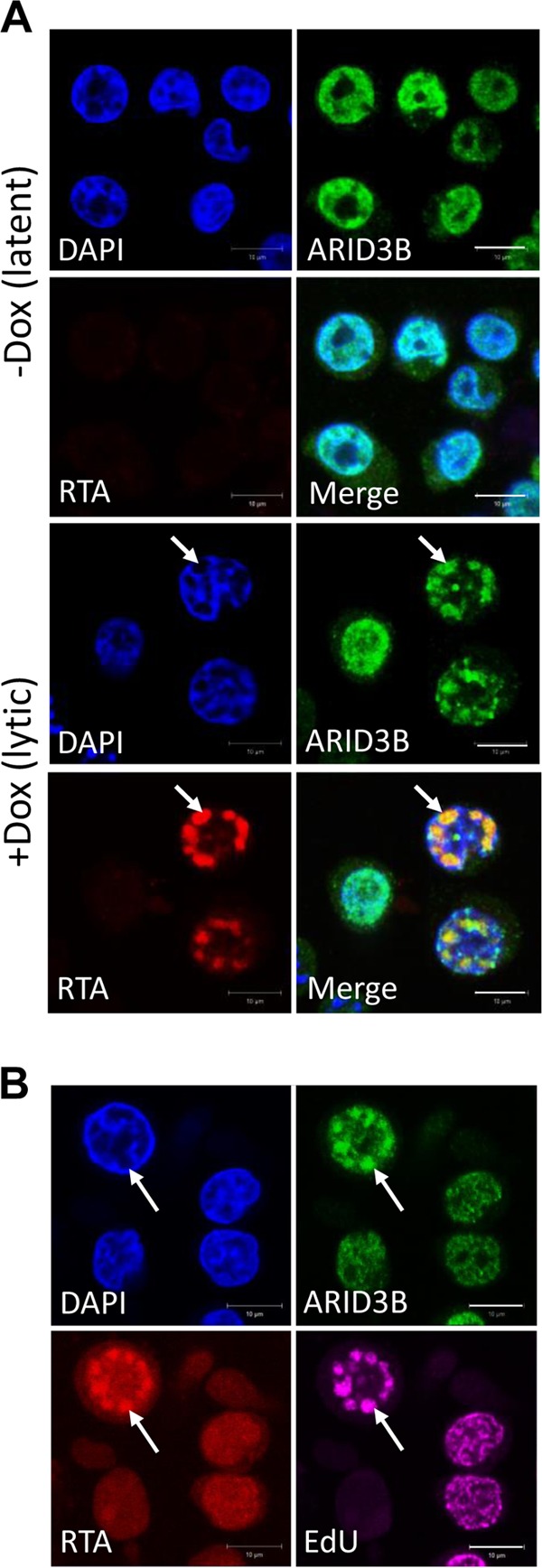
ARID3B relocalizes to KSHV replication compartments upon reactivation of the lytic cycle. (A) Confocal immunofluorescence analysis demonstrated that ARID3B relocalized to discrete, RTA-positive foci that resemble replication compartments (white arrows) upon doxycycline (Dox)-induced reactivation of the KSHV lytic cycle in TREx-BCBL1-RTA cells. (B) Further evidence suggesting that ARID3B relocalized to replication compartments, demonstrated by its colocalization with RTA and EdU-positive foci (white arrows). EdU is used as a marker of newly replicated virus (57). Bar, 10 μm.

Given that ARID3B expression levels were influenced by RTA expression and lytic reactivation, we next asked whether it was involved in regulating the KSHV lytic cycle. Using an immunofluorescence microscopy approach, we showed that in latently infected TREx-BCBL-1-RTA cells, ARID3B expression displayed a pan-nuclear (excluding nucleolus) localization [Fig. 3A, -Dox (latent)]. However, upon lytic reactivation, ARID3B is relocalized to discrete, RTA-positive foci, with close proximity to the nuclear periphery [Fig. 3A, +Dox (lytic); compare the RTA-positive, reactivated cells to the RTA-negative cell in the same field of view], reminiscent of replication compartments (also known as replication and transcription compartments [RTCs]). Replication compartments are distinct, RTA-positive foci found at the nuclear periphery associated with newly replicated viral DNA (which is observed via incorporation of bromodeoxyuridine [BrdU] or EdU that specifically marks viral DNA due to KSHV's ability to block cellular DNA replication during reactivation). We performed an independent experiment to ask if ARID3B did indeed relocalize to these foci. Consequently, ARID3B was again shown to costain with RTA and to colocalize with EdU-positive foci (Fig. 3B), suggesting that it did indeed relocalize from a pan-nuclear pattern into KSHV replication compartments upon lytic reactivation.

### ARID3B modulates the KSHV lytic cycle.

**FIG 4:**
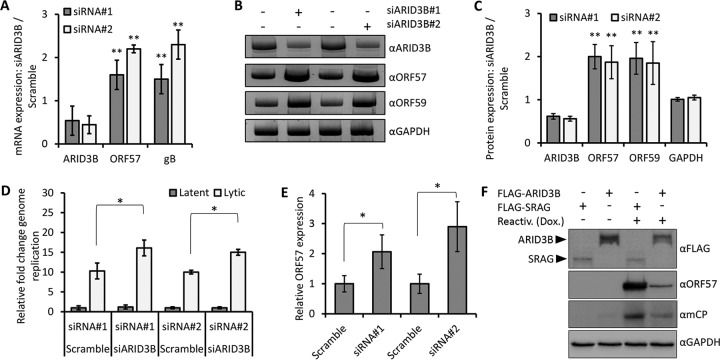
ARID3B inhibits KSHV lytic reactivation. (A) Latently infected iSLK-BAC16 cells were independently transfected with two separate siRNA pools targeting *ARID3B* (or a nontargeting scramble control siRNA) for 72 h and then reactivated by addition of doxycycline (a further 24 h; see Materials and Methods). RT-qPCR was used to quantify *ARID3B*, *ORF57*, and *gB* levels, and the fold difference in expression was compared to scramble control using *GAPDH* levels to normalize between samples. Error bars represent standard deviations from the mean from two independent experiments, with mean values calculated from the technical replicates of each experiment. Statistical analyses demonstrated a significant increase in lytic gene expression in ARID3B siRNA-treated cells compared to scramble control. **, *P* < 0.0001 (Student's *t* test). (B) Knockdown of ARID3B led to an enhancement of lytic protein expression. (C) Quantification of panel B (see Materials and Methods). (D) Knockdown of ARID3B enhanced viral genome replication. ARID3B siRNA or scramble control siRNA transfection of iSLK-BAC16 cells followed by reactivation of the lytic cycle for 72 h led to an increase in viral genome replication, as quantified by qPCR of the KSHV *ORF57* gene. Cellular GAPDH was used to normalize between samples, and error bars represent standard deviations from the mean from three independent biological replicates (*, *P* < 0.05). (E) Knockdown of ARID3B enhances virion production. Media from iSLK-BAC16 cells that had been transfected with ARID3B siRNA or scramble control siRNAs were used to infect KSHV-negative HEK293T cells. As a measure of infection, RT-qPCR analysis of the KSHV lytic gene *ORF57* was performed 24 h following infection. Data represent three independent infections in a single experiment, and Student's *t* test was used to determine statistical significance (*, *P* < 0.05). (F) Overexpression of FLAG-ARID3B inhibits reactivation of lytic cycle-associated protein expression. Expression vector containing FLAG-tagged ARID3B (FLAG-ARID3B) or FLAG-SRAG (used as a negative control) was transfected into iSLK-BAC16 cells for 24 h, followed by doxycycline (Dox.)-induced reactivation of the KSHV lytic cycle. Cell lysates were harvested 24 h later and subjected to immunoblot analysis of lytic proteins ORF57 and mCP (minor capsid protein).

To investigate if ARID3B influenced the KSHV lytic cycle, we transfected iSLK-BAC16 cells with two separate pools of *ARID3B*-targeted or scramble control siRNAs and induced the lytic cycle with doxycycline for 24 h. Given that ARID3B has been suggested to function as a transcription factor, we anticipated that it might be important for enhancing RTA-mediated expression of lytic genes. Using RT-qPCR, we demonstrated an approximately 50% knockdown of *ARID3B*. This was sufficient to enhance lytic gene expression between 1.5- and 2.5-fold, as shown for lytic genes *ORF57* and *gB* (Fig. 4A), suggesting that ARID3B might actually inhibit lytic gene expression. We noticed that the two siRNAs had slightly different effects on lytic gene expression; these two siRNA preparations were mixtures of three different sequences, and it might be that one of these more potently targets *ARID3B*. However, we cannot fully explain this at the current time, and this difference was not observed in other assays with this set of siRNAs. ARID3B knockdown was also associated with an approximately 2-fold increase in lytic protein expression (Fig. 4B and C). Accordingly, ARID3B knockdown, followed by reactivation of the lytic cycle, also led to a significant enhancement of KSHV genome replication compared to cells transfected with a scramble control siRNA (Fig. 4D). Importantly, knockdown in latently infected cells did not activate viral DNA replication (Fig. 4D), suggesting that RTA expression or the lytic cycle was required to activate ARID3B's inhibitory properties.

To ask if the increases in lytic cycle-associated expression and genome replication led to an enhanced productive infection, we harvested and clarified medium from iSLK-BAC16 cells that had been transfected with ARID3B siRNA and reactivated with doxycycline. We transferred this medium to KSHV-negative cells (HEK293T) and, 24 h later, purified total cellular RNA. Using RT-qPCR analysis of the KSHV *ORF57* gene as a measure of virus infection, we demonstrated that knockdown of ARID3B led to an enhancement of virus infection, suggesting that ARID3B knockdown led to an increase of released virions (Fig. 4E). Together, these data showed that ARID3B knockdown enhanced the KSHV lytic cycle, contrary to our initial hypothesis. However, these differences were modest given our inability to reduce ARID3B expression more than 50 to 60%. Therefore, we took an alternative approach and asked if ARID3B overexpression could inhibit KSHV reactivation. Here, we showed that transfection of FLAG-tagged ARID3B, but not FLAG-SRAG (used as a control because we know that its expression does not modulate lytic reactivation), in iSLK-BAC16 cells for 24 h, followed by reactivation of the lytic cycle, inhibited the expression of KSHV lytic genes (Fig. 4F). Together, these data demonstrate that ARID3B is able to modulate KSHV reactivation, and not enhance it, as originally predicted.

### ARID3B interacts with an A/T-rich region of oriLyt in a lytic reactivation-dependent manner.

**FIG 5:**
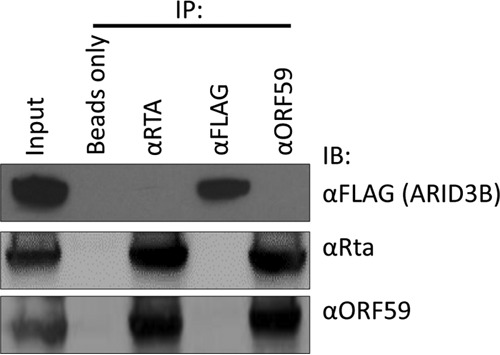
RTA and ARID3B do not directly interact. Immunoprecipitation assay showing that RTA interacts with known binding partner ORF59 but not ARID3B. The lytic cycle was induced in ARID3B-transfected iSLK-BAC16 cells. Proteins were immunoprecipitated with the indicated antibodies, followed by immunoblot (IB) analysis.

**FIG 6:**
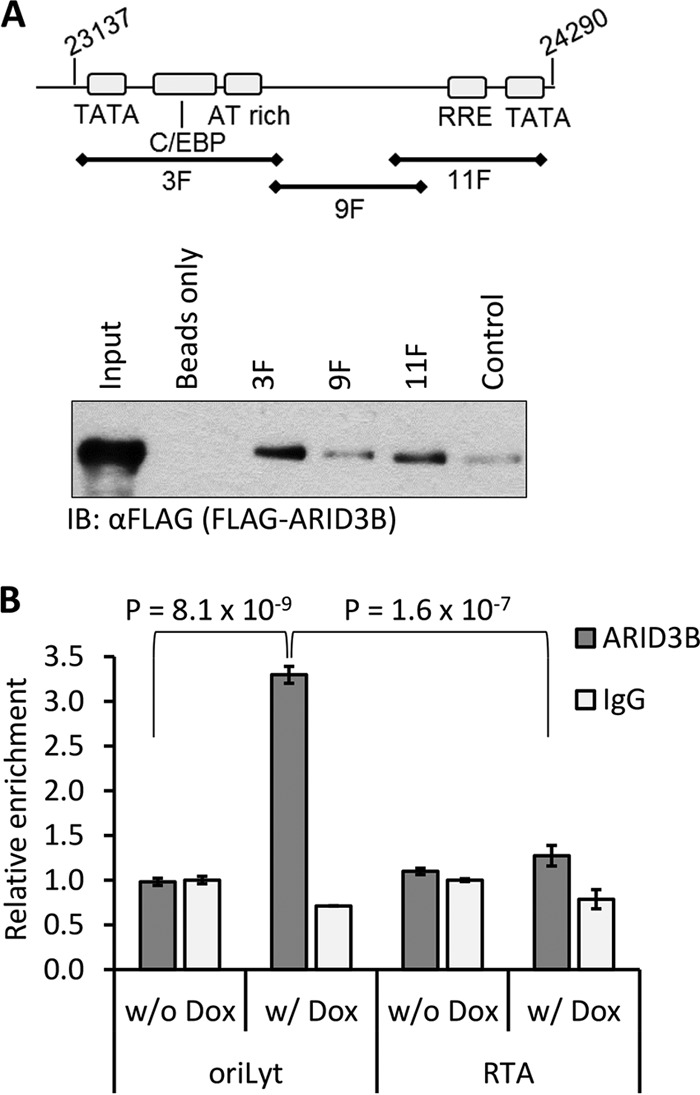
ARID3B interacts with the KSHV genome in a lytic reactivation-dependent manner. (A) DNA affinity assays performed according to the method in reference 25; biotinylated PCR products spanning oriLyt left of KSHV (GenBank accession number NC_009333.1) and control DNA amplified from the RTA coding region were bound to streptavidin-Dynabeads and incubated with lysates from reactivated 293T rKSHV.219 cells expressing FLAG-ARID3B. This suggested that ARID3B bound these sequences with preference for the A/T-rich region. IB, immunoblotting. (B) TREx-BCBL1-RTA cells were treated with doxycycline for 18 h (w/Dox) to reactivate the lytic cycle or were left untreated (w/o Dox). Samples were subjected to ChIP with an antibody to ARID3B or normal rabbit IgG and primers specific for sequences in the A/T-rich region of oriLyt or the RTA coding region. The percentage of output versus input DNA was calculated and is presented relative to normal rabbit IgG values from unreactivated (w/o Dox) cells (set to 1). Data represent two biological replicates (i.e., independent ChIPs) and two technical replicates per ChIP from a single experiment, and values are given as the mean ± standard deviation. Student *t* tests were used to determine statistical significance.

To investigate a potential mechanism whereby ARID3B tempered KSHV lytic reactivation, we tested the possibility that ARID3B interacted with RTA and inhibited its function. RTA interacts with various cellular proteins that are known to be inhibitory, including PARP-1 (34), TLE2 (33), and HDAC1 (35). However, coimmunoprecipitation assays demonstrated that while RTA interacted with a known binding partner, ORF59 (22), it did not interact with ARID3B (Fig. 5). Next, we reasoned that ARID3B, as a DNA binding protein, might interact with viral genomes. Furthermore, due to its predicted preference for A/T-rich sequences, and the existence of these sequences in oriLyt, we focused our attention on this region of the KSHV genome. We performed a DNA affinity assay that has previously been reported as a method for identifying proteins involved in KSHV reactivation (25). Biotinylated PCR products that spanned oriLyt were amplified from KSHV DNA (Fig. 6A), bound to streptavidin-coated beads, and incubated with lysate from reactivated HEK293T-rKSHV.219 cells that had been transfected with FLAG-ARID3B 24 h earlier. Compared to control DNA (amplified from the RTA coding region) and a central G/C-rich repeat region (9F), we found that ARID3B preferentially bound to region 3F, which contains the A/T-rich region of KSHV oriLyt (Fig. 6A). ARID3B also bound to region 11F, which contained the RTA-responsive element (RRE), although this appeared to be slightly reduced compared to the 3F region. Interestingly, it was shown previously that many host and viral proteins involved in KSHV reactivation specifically bind these two regions in oriLyt (25).

We confirmed this result using chromatin immunoprecipitation (ChIP) assays using TREx-BCBL-1-RTA cells. This allowed to us investigate whether ARID3B interacted with the KSHV genome in infected cells, in addition to asking if this was linked with reactivation of the lytic cycle. Using an antibody that has previously been used in ChIP assays (43), we showed that endogenous ARID3B interacted preferentially with the A/T-rich region of oriLyt (3.5-fold enrichment over control IgG antibody) and not within the RTA gene (Fig. 6B). Furthermore, we showed that this interaction was dependent on reactivation of the KSHV lytic cycle, as no binding (above control IgG antibody) was observed in latent cells (Fig. 6B). These data, together with data in Fig. 2 (lytic cycle-associated relocalization) and Fig. 4 (knockdown of ARID3B does not induce viral DNA replication), suggest that ARID3B influences KSHV biology only upon reactivation of the lytic cycle.

## DISCUSSION

It is well established that KSHV is the etiologic agent of various malignancies in immunocompromised individuals, particularly those infected with HIV. Indeed, KS is the most prevalent malignancy in regions with endemic HIV and, for HIV sufferers, is one of a number of diseases that are used as a guideline for the diagnosis of AIDS (i.e., KS is considered an AIDS-defining disease) (2). Importantly, various reports, including data from clinical studies, link reactivation of the KSHV lytic cycle with the pathogenesis of KS. Therefore, it is imperative that we understand the mechanisms that regulate this aspect of KSHV biology. RTA, the KSHV lytic switch protein, is both necessary and sufficient for reactivation of the lytic cycle (8). While we have a relatively good understanding of how RTA drives the viral transcriptional program responsible for lytic reactivation (8), how RTA influences cellular responses is less well understood. In this report, we developed a cell line with inducible RTA expression and, by applying a quantitative proteomics approach, used it to investigate what impact RTA expression has on the cellular proteome.

We identified a number of cellular pathways that were associated with RTA expression, and some of these are the focus of current research. Of note, ubiquitin-mediated proteolysis was one of the gene ontology terms included in our bioinformatics analyses. We, and others, have recently reported the importance of this pathway for regulating herpesvirus gene expression and demonstrated that this pathway represents a potential therapeutic target for the treatment of herpesvirus disease (62, 72). In addition to studying cellular pathways, we generated a list of the top 15 proteins based on increased protein abundance in RTA-expressing cells. In this report, we focused on a little-known protein, ARID3B. We initially hypothesized that, as a prosurvival transcription factor (43, 44), this protein would cooperate with RTA during reactivation of the lytic cycle. However, we actually found that ARID3B played an inhibitory role, and its expression was associated with modulating lytic reactivation.

A hallmark of all herpesviruses is their ability to establish a latent infection characterized by a highly restricted gene expression program. This is an incredibly effective means of evading detection by the immune system and ensures that infection is maintained throughout the life of the host. Nevertheless, periodic reactivation of the lytic cycle is necessary in order to maintain the population of infected cells. However, the lytic cycle must be exquisitely regulated so that it does not lead to uncontrolled virion production and severe disease in its host. We observed that upon RTA expression, or reactivation of the lytic cycle, ARID3B expression was increased at both the mRNA and protein levels. To ascertain if this had functional consequences for KSHV reactivation, we performed a series of experiments that showed that ARID3B was able to block reactivation. This included siRNA-mediated knockdown of ARID3B, which resulted in enhanced lytic gene expression and lytic protein expression and an increase in genome replication. Furthermore, knockdown of ARID3B potentially led to an increase in virion production, as suggested by reinfection assays. As an alternative approach, overexpression of ARID3B significantly abrogated lytic protein expression. Importantly, knockdown of ARID3B in latently infected cells did not reactivate the lytic cycle, suggesting that it was not involved in the maintenance of latency. Therefore, RTA-mediated activation of ARID3B specifically served to regulate lytic reactivation; however, it is also possible that this inhibitory function may be important during the establishment phase of latency.

KSHV reactivation leads to the upregulation of several negative feedback processes that temper the lytic cycle, many of which center on abrogating RTA's interaction with RBP-Jκ (required for transactivating various lytic gene promoters via the “indirect” mechanism [8]). For example, and in a similar vein as the observed RTA-enhanced ARID3B expression, RTA enhances the expression of the corepressor transducin-like enhancer of split 2 (TLE2), which blocked lytic reactivation by competing with RBP-Jκ for the same binding site in RTA (33) (we did not identify TLE2 in our data sets). Lytic reactivation also promotes nuclear translocation of the transcription factor NF-κB, which in turn negatively regulates lytic cycle-associated gene expression by competing with RTA for RBP-Jκ binding (32, 73, 74). Furthermore, activated NF-κB is required for the expression of latency-associated genes (e.g., *LANA*, *vFLIP*, and *vCyclin*), and LANA has been shown to inhibit lytic reactivation, again, by interfering with RTA–RBP-Jκ interactions (75, 76). Additional negative regulatory mechanisms involve RTA interactions with histone deacetylase enzymes HDAC1 (35) and SIRT1 (77), which limits RTA's ability to activate lytic gene transcription. We do not currently know how RTA led to increases in ARID3B expression. As a transcription factor, RTA may drive the expression of *ARID3B*; it may also be possible that RTA induces ARID3B expression indirectly. Here, two possibilities exist: (i) RTA expression may promote a cellular response that is linked to ARID3B expression or (ii) RTA may sequester a repressor that would normally silence *ARID3B*. As there are no reports describing the regulation of ARID3B expression, it is not possible to go beyond speculation.

Commencement of the lytic cycle leads to a dramatic remodeling of the cell nucleus and the formation of discrete foci (termed replication compartments) where viral transcription, genome replication, and capsid assembly take place (78). For KSHV, the formation of replication compartments was recently shown to be dependent on the chaperone function of Hsp70 proteins (57). We observed that, upon lytic reactivation, ARID3B relocalized from a pan-nuclear pattern to replication compartments as shown by its colocalization with RTA and EdU. Of note, it was also recently shown that lytic reactivation induced the relocalization of TLE2 into replication compartments (33), highlighting a common feature among negative regulators of the lytic cycle. This suggested that ARID3B might inhibit reactivation by interacting with a protein required for lytic reactivation (cellular or viral), or the viral genome, although these scenarios are not necessarily mutually exclusive. Interestingly, RTA and ARID3B did not interact. This was somewhat surprising; however, as ARID3B is a DNA binding protein, an interaction with RTA may not be necessary for it to associate with reactivated KSHV. Therefore, unlike the majority of lytic cycle regulators that have been shown to interact directly with RTA, ARID3B performs this task via a different mechanism.

We reasoned that ARID3B, as a DNA binding protein, might interact with viral genomes. Particularly, given its proposed preference for A/T-rich sequences and the presence of these sequences in oriLyt, we considered the possibility that ARID3B binds to this region of the genome. Indeed, DNA affinity (Fig. 6A) and ChIP (Fig. 6B) assays demonstrated that this was the case. The DNA affinity assay showed us that overexpressed ARID3B preferentially bound to a region of oriLyt that also contains A/T-rich palindromic sequences in addition to a downstream region which contained the RTA-responsive element (RRE). Interestingly, it was previously shown that many host and viral proteins involved in KSHV reactivation specifically bind these two regions in oriLyt (25).

**FIG 7:**
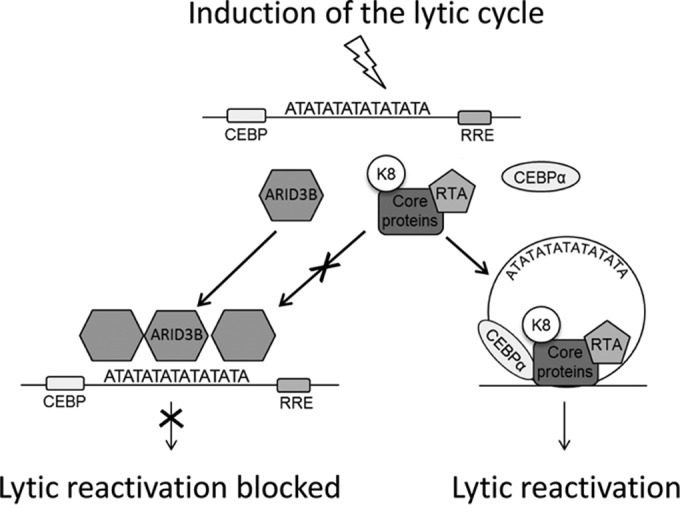
A model of how ARID3B inhibits lytic reactivation. Periodic reactivation of KSHV involves replication of the viral genome and the production of new virions, a necessary step for the maintenance of latency and the pathogenesis of KS (2). Viral DNA replication initiates from the *cis*-acting oriLyt where proteins essential for this process are recruited. These include viral proteins RTA and K8 and the six core proteins (DNA polymerase, DNA processivity factor, etc.) (80) in addition to various cellular *trans*-acting proteins (25). We hypothesize that during reactivation RTA is expressed, leading to the enhanced expression of ARID3B and its recruitment to viral genomes, specifically AT-rich elements such as those found in oriLyt. ARID3B may compete with factors required for reactivation for binding with the KSHV genome in order to modulate the levels of reactivation or for the establishment of latency.

Importantly, ChIP assays not only allowed us to confirm binding *in vivo*, but they also allowed us to ask if ARID3B's interaction was dependent on lytic reactivation. This was an important question as much of our data suggested that ARID3B's inhibitory properties were associated with RTA expression, which is silenced during latency; for example, and as mentioned above, knockdown of ARID3B from latently infected cells did not appear to stimulate lytic gene expression (suggesting that it does not have a role in the maintenance of latency). Strikingly, this interaction was indeed dependent on reactivation of the lytic cycle, as no binding was observed on the latent genome. This might suggest that the chromatinized latent KSHV genome precludes ARID3B access; however, upon reactivation, which is associated with removal of nucleosomes, ARID3B can bind. However, it is not currently known if ARID3B binding is dependent on chromatin status of DNA. Additionally, whether this is dependent on direct DNA binding or requires PPIs and whether ARID3B blocks transcription or DNA replication are still open questions. Nevertheless, these data provide weight to the hypothesis that ARID3B specifically responds to reactivation of the lytic cycle from latency and inhibits it via interactions with the KSHV genome. We propose that this interaction likely competes with oriLyt-associated factors required for reactivation of the lytic cycle (Fig. 7).

We were surprised to observe that RTA expression induced a number of proteins and pathways associated with transcriptional silencing (Tables 1 and 2). Although it will be critical to validate these observations, this might not be surprising given that *de novo* KSHV infection of tissue culture cells leads to viral latency, despite an initial burst of lytic cycle-associated gene expression (79). Therefore, while RTA drives expression from the viral genome during reactivation, it may also induce a number of processes (directly or indirectly) that ultimately silence lytic gene expression.

In summary, we have identified a novel cellular protein, ARID3B, which responds to the lytic cycle of KSHV and functions to inhibit it. Lytic cycle-associated induction of negative feedback mechanisms is clearly an important feature of herpesvirus biology and is consistent with a virus that favors lifelong infection of its host.

## References

[1] Dittmer DP, Damania B 2013 Kaposi sarcoma associated herpesvirus pathogenesis (KSHV)—an update. Curr Opin Virol 3:238–244. doi:10.1016/j.coviro.2013.05.012. 23769237 PMC3716290

[2] Mesri EA, Cesarman E, Boshoff C 2010 Kaposi's sarcoma and its associated herpesvirus. Nat Rev Cancer 10:707–719. doi:10.1038/nrc2888. 20865011 PMC4721662

[3] Godfrey A, Anderson J, Papanastasiou A, Takeuchi Y, Boshoff C 2005 Inhibiting primary effusion lymphoma by lentiviral vectors encoding short hairpin RNA. Blood 105:2510–2518. doi:10.1182/blood-2004-08-3052. 15572586

[4] Ambroziak JA, Blackbourn DJ, Herndier BG, Glogau RG, Gullett JH, McDonald AR, Lennette ET, Levy JA 1995 Herpes-like sequences in HIV-infected and uninfected Kaposi's sarcoma patients. Science 268:582–583. doi:10.1126/science.7725108. 7725108

[5] Quinlivan EB, Zhang C, Stewart PW, Komoltri C, Davis MG, Wehbie RS 2002 Elevated virus loads of Kaposi's sarcoma-associated human herpesvirus 8 predict Kaposi's sarcoma disease progression, but elevated levels of human immunodeficiency virus type 1 do not. J Infect Dis 185:1736–1744. doi:10.1086/340652. 12085319

[6] Campbell TB, Borok M, Gwanzura L, MaWhinney S, White IE, Ndemera B, Gudza I, Fitzpatrick L, Schooley RT 2000 Relationship of human herpesvirus 8 peripheral blood virus load and Kaposi's sarcoma clinical stage. AIDS 14:2109–2116. doi:10.1097/00002030-200009290-00006. 11061651

[7] Martin DF, Kuppermann BD, Wolitz RA, Palestine AG, Li H, Robinson CA 1999 Oral ganciclovir for patients with cytomegalovirus retinitis treated with a ganciclovir implant. Roche Ganciclovir Study Group. N Engl J Med 340:1063–1070. 10194235 10.1056/NEJM199904083401402

[8] Guito J, Lukac DM 2012 KSHV Rta promoter specification and viral reactivation. Front Microbiol 3:30. doi:10.3389/fmicb.2012.00030. 22347875 PMC3278982

[9] Dalton-Griffin L, Wilson SJ, Kellam P 2009 X-box binding protein 1 contributes to induction of the Kaposi's sarcoma-associated herpesvirus lytic cycle under hypoxic conditions. J Virol 83:7202–7209. doi:10.1128/JVI.00076-09. 19403667 PMC2704782

[10] Yu F, Feng J, Harada JN, Chanda SK, Kenney SC, Sun R 2007 B cell terminal differentiation factor XBP-1 induces reactivation of Kaposi's sarcoma-associated herpesvirus. FEBS Lett 581:3485–3488. doi:10.1016/j.febslet.2007.06.056. 17617410

[11] Davis DA, Rinderknecht AS, Zoeteweij JP, Aoki Y, Read-Connole EL, Tosato G, Blauvelt A, Yarchoan R 2001 Hypoxia induces lytic replication of Kaposi sarcoma-associated herpesvirus. Blood 97:3244–3250. doi:10.1182/blood.V97.10.3244. 11342455

[12] Bu W, Carroll KD, Palmeri D, Lukac DM 2007 Kaposi's sarcoma-associated herpesvirus/human herpesvirus 8 ORF50/Rta lytic switch protein functions as a tetramer. J Virol 81:5788–5806. doi:10.1128/JVI.00140-07. 17392367 PMC1900300

[13] Tsai WH, Wang PW, Lin SY, Wu IL, Ko YC, Chen YL, Li M, Lin SF 2012 Ser-634 and Ser-636 of Kaposi's sarcoma-associated herpesvirus RTA are involved in transactivation and are potential Cdk9 phosphorylation sites. Front Microbiol 3:60. doi:10.3389/fmicb.2012.00060. 22371709 PMC3283893

[14] Sun R, Lin SF, Gradoville L, Yuan Y, Zhu F, Miller G 1998 A viral gene that activates lytic cycle expression of Kaposi's sarcoma-associated herpesvirus. Proc Natl Acad Sci U S A 95:10866–10871. doi:10.1073/pnas.95.18.10866. 9724796 PMC27987

[15] Chang PJ, Shedd D, Miller G 2005 Two subclasses of Kaposi's sarcoma-associated herpesvirus lytic cycle promoters distinguished by open reading frame 50 mutant proteins that are deficient in binding to DNA. J Virol 79:8750–8763. doi:10.1128/JVI.79.14.8750-8763.2005. 15994769 PMC1168723

[16] Song MJ, Brown HJ, Wu TT, Sun R 2001 Transcription activation of polyadenylated nuclear RNA by RTA in human herpesvirus 8/Kaposi's sarcoma-associated herpesvirus. J Virol 75:3129–3140. doi:10.1128/JVI.75.7.3129-3140.2001. 11238840 PMC114107

[17] Chang PJ, Shedd D, Gradoville L, Cho MS, Chen LW, Chang J, Miller G 2002 Open reading frame 50 protein of Kaposi's sarcoma-associated herpesvirus directly activates the viral PAN and K12 genes by binding to related response elements. J Virol 76:3168–3178. doi:10.1128/JVI.76.7.3168-3178.2002. 11884541 PMC136055

[18] Zhang J, Wang J, Wood C, Xu D, Zhang L 2005 Kaposi's sarcoma-associated herpesvirus/human herpesvirus 8 replication and transcription activator regulates viral and cellular genes via interferon-stimulated response elements. J Virol 79:5640–5652. doi:10.1128/JVI.79.9.5640-5652.2005. 15827179 PMC1082735

[19] Liang Y, Ganem D 2003 Lytic but not latent infection by Kaposi's sarcoma-associated herpesvirus requires host CSL protein, the mediator of Notch signaling. Proc Natl Acad Sci U S A 100:8490–8495. doi:10.1073/pnas.1432843100. 12832621 PMC166256

[20] Matsumura S, Fujita Y, Gomez E, Tanese N, Wilson AC 2005 Activation of the Kaposi's sarcoma-associated herpesvirus major latency locus by the lytic switch protein RTA (ORF50). J Virol 79:8493–8505. doi:10.1128/JVI.79.13.8493-8505.2005. 15956592 PMC1143749

[21] Palmeri D, Carroll KD, Gonzalez-Lopez O, Lukac DM 2011 Kaposi's sarcoma-associated herpesvirus Rta tetramers make high-affinity interactions with repetitive DNA elements in the Mta promoter to stimulate DNA binding of RBP-Jk/CSL. J Virol 85:11901–11915. doi:10.1128/JVI.05479-11. 21880753 PMC3209305

[22] Rossetto CC, Susilarini NK, Pari GS 2011 Interaction of Kaposi's sarcoma-associated herpesvirus ORF59 with oriLyt is dependent on binding with K-Rta. J Virol 85:3833–3841. doi:10.1128/JVI.02361-10. 21289111 PMC3126130

[23] Wang Y, Li H, Chan MY, Zhu FX, Lukac DM, Yuan Y 2004 Kaposi's sarcoma-associated herpesvirus ori-Lyt-dependent DNA replication: cis-acting requirements for replication and ori-Lyt-associated RNA transcription. J Virol 78:8615–8629. doi:10.1128/JVI.78.16.8615-8629.2004. 15280471 PMC479094

[24] Wang Y, Tang Q, Maul GG, Yuan Y 2006 Kaposi's sarcoma-associated herpesvirus ori-Lyt-dependent DNA replication: dual role of replication and transcription activator. J Virol 80:12171–12186. doi:10.1128/JVI.00990-06. 17020951 PMC1676287

[25] Wang Y, Li H, Tang Q, Maul GG, Yuan Y 2008 Kaposi's sarcoma-associated herpesvirus ori-Lyt-dependent DNA replication: involvement of host cellular factors. J Virol 82:2867–2882. doi:10.1128/JVI.01319-07. 18199640 PMC2259006

[26] Gunther T, Grundhoff A 2010 The epigenetic landscape of latent Kaposi sarcoma-associated herpesvirus genomes. PLoS Pathog 6:e1000935. doi:10.1371/journal.ppat.1000935. 20532208 PMC2880564

[27] Gunther T, Schreiner S, Dobner T, Tessmer U, Grundhoff A 2014 Influence of ND10 components on epigenetic determinants of early KSHV latency establishment. PLoS Pathog 10:e1004274. doi:10.1371/journal.ppat.1004274. 25033267 PMC4102598

[28] Toth Z, Maglinte DT, Lee SH, Lee HR, Wong LY, Brulois KF, Lee S, Buckley JD, Laird PW, Marquez VE, Jung JU 2010 Epigenetic analysis of KSHV latent and lytic genomes. PLoS Pathog 6:e1001013. doi:10.1371/journal.ppat.1001013. 20661424 PMC2908616

[29] Lan K, Kuppers DA, Verma SC, Robertson ES 2004 Kaposi's sarcoma-associated herpesvirus-encoded latency-associated nuclear antigen inhibits lytic replication by targeting Rta: a potential mechanism for virus-mediated control of latency. J Virol 78:6585–6594. doi:10.1128/JVI.78.12.6585-6594.2004. 15163750 PMC416549

[30] Li Q, Zhou F, Ye F, Gao SJ 2008 Genetic disruption of KSHV major latent nuclear antigen LANA enhances viral lytic transcriptional program. Virology 379:234–244. doi:10.1016/j.virol.2008.06.043. 18684478 PMC2626151

[31] Bellare P, Ganem D 2009 Regulation of KSHV lytic switch protein expression by a virus-encoded microRNA: an evolutionary adaptation that fine-tunes lytic reactivation. Cell Host Microbe 6:570–575. doi:10.1016/j.chom.2009.11.008. 20006845 PMC2822622

[32] Izumiya Y, Izumiya C, Hsia D, Ellison TJ, Luciw PA, Kung HJ 2009 NF-kappaB serves as a cellular sensor of Kaposi's sarcoma-associated herpesvirus latency and negatively regulates K-Rta by antagonizing the RBP-Jkappa coactivator. J Virol 83:4435–4446. doi:10.1128/JVI.01999-08. 19244329 PMC2668470

[33] He Z, Liu Y, Liang D, Wang Z, Robertson ES, Lan K 2010 Cellular corepressor TLE2 inhibits replication-and-transcription-activator-mediated transactivation and lytic reactivation of Kaposi's sarcoma-associated herpesvirus. J Virol 84:2047–2062. doi:10.1128/JVI.01984-09. 19939918 PMC2812399

[34] Gwack Y, Nakamura H, Lee SH, Souvlis J, Yustein JT, Gygi S, Kung HJ, Jung JU 2003 Poly(ADP-ribose) polymerase 1 and Ste20-like kinase hKFC act as transcriptional repressors for gamma-2 herpesvirus lytic replication. Mol Cell Biol 23:8282–8294. doi:10.1128/MCB.23.22.8282-8294.2003. 14585985 PMC262387

[35] Gwack Y, Byun H, Hwang S, Lim C, Choe J 2001 CREB-binding protein and histone deacetylase regulate the transcriptional activity of Kaposi's sarcoma-associated herpesvirus open reading frame 50. J Virol 75:1909–1917. doi:10.1128/JVI.75.4.1909-1917.2001. 11160690 PMC115137

[36] Griffiths DA, Abdul-Sada H, Knight LM, Jackson BR, Richards K, Prescott EL, Peach AH, Blair GE, Macdonald A, Whitehouse A 2013 Merkel cell polyomavirus small T antigen targets the NEMO adaptor protein to disrupt inflammatory signaling. J Virol 87:13853–13867. doi:10.1128/JVI.02159-13. 24109239 PMC3838273

[37] Jackson BR, Noerenberg M, Whitehouse A 2014 A novel mechanism inducing genome instability in Kaposi's sarcoma-associated herpesvirus infected cells. PLoS Pathog 10:e1004098. doi:10.1371/journal.ppat.1004098. 24788796 PMC4006916

[38] Knight LM, Stakaityte G, Wood JJ, Abdul-Sada H, Griffiths DA, Howell GJ, Wheat R, Blair GE, Steven NM, Macdonald A, Blackbourn DJ, Whitehouse A 2015 Merkel cell polyomavirus small T antigen mediates microtubule destabilization to promote cell motility and migration. J Virol 89:35–47. doi:10.1128/JVI.02317-14. 25320307 PMC4301106

[39] Munday DC, Surtees R, Emmott E, Dove BK, Digard P, Barr JN, Whitehouse A, Matthews D, Hiscox JA 2012 Using SILAC and quantitative proteomics to investigate the interactions between viral and host proteomes. Proteomics 12:666–672. doi:10.1002/pmic.201100488. 22246955

[40] Owen CB, Hughes DJ, Baquero-Perez B, Berndt A, Schumann S, Jackson BR, Whitehouse A 2014 Utilising proteomic approaches to understand oncogenic human herpesviruses (review). Mol Clin Oncol 2:891–903. 25279171 10.3892/mco.2014.341 PMC4179824

[41] Herrscher RF, Kaplan MH, Lelsz DL, Das C, Scheuermann R, Tucker PW 1995 The immunoglobulin heavy-chain matrix-associating regions are bound by Bright: a B cell-specific trans-activator that describes a new DNA-binding protein family. Genes Dev 9:3067–3082. doi:10.1101/gad.9.24.3067. 8543152

[42] Rhee C, Lee BK, Beck S, Anjum A, Cook KR, Popowski M, Tucker HO, Kim J 2014 Arid3a is essential to execution of the first cell fate decision via direct embryonic and extraembryonic transcriptional regulation. Genes Dev 28:2219–2232. doi:10.1101/gad.247163.114. 25319825 PMC4201284

[43] Bobbs A, Gellerman K, Hallas WM, Joseph S, Yang C, Kurkewich J, Cowden Dahl KD 2015 ARID3B directly regulates ovarian cancer promoting genes. PLoS One 10:e0131961. doi:10.1371/journal.pone.0131961. 26121572 PMC4486168

[44] Roy L, Samyesudhas SJ, Carrasco M, Li J, Joseph S, Dahl R, Cowden Dahl KD 2014 ARID3B increases ovarian tumor burden and is associated with a cancer stem cell gene signature. Oncotarget 5:8355–8366. doi:10.18632/oncotarget.2247. 25327563 PMC4226688

[45] Casanova JC, Uribe V, Badia-Careaga C, Giovinazzo G, Torres M, Sanz-Ezquerro JJ 2011 Apical ectodermal ridge morphogenesis in limb development is controlled by Arid3b-mediated regulation of cell movements. Development 138:1195–1205. doi:10.1242/dev.057570. 21307092

[46] Takebe A, Era T, Okada M, Martin Jakt L, Kuroda Y, Nishikawa S 2006 Microarray analysis of PDGFR alpha+ populations in ES cell differentiation culture identifies genes involved in differentiation of mesoderm and mesenchyme including ARID3b that is essential for development of embryonic mesenchymal cells. Dev Biol 293:25–37. doi:10.1016/j.ydbio.2005.12.016. 16530748

[47] Akhavantabasi S, Sapmaz A, Tuna S, Erson-Bensan AE 2012 miR-125b targets ARID3B in breast cancer cells. Cell Struct Funct 37:27–38. doi:10.1247/csf.11025. 22307404

[48] Joseph S, Deneke VE, Cowden Dahl KD 2012 ARID3B induces tumor necrosis factor alpha mediated apoptosis while a novel ARID3B splice form does not induce cell death. PLoS One 7:e42159. doi:10.1371/journal.pone.0042159. 22860069 PMC3409141

[49] Kobayashi K, Era T, Takebe A, Jakt LM, Nishikawa S 2006 ARID3B induces malignant transformation of mouse embryonic fibroblasts and is strongly associated with malignant neuroblastoma. Cancer Res 66:8331–8336. doi:10.1158/0008-5472.CAN-06-0756. 16951138

[50] Kobayashi K, Jakt LM, Nishikawa SI 2013 Epigenetic regulation of the neuroblastoma genes, Arid3b and Mycn. Oncogene 32:2640–2648. doi:10.1038/onc.2012.285. 22751132 PMC3664305

[51] Oguz Erdogan AS, Ozdemirler N, Oyken M, Alper M, Erson-Bensan AE 2014 ARID3B expression in primary breast cancers and breast cancer-derived cell lines. Cell Oncol (Dordr) 37:289–296. doi:10.1007/s13402-014-0185-5. 25120063 PMC13004475

[52] Samyesudhas SJ, Roy L, Cowden Dahl KD 2014 Differential expression of ARID3B in normal adult tissue and carcinomas. Gene 543:174–180. doi:10.1016/j.gene.2014.04.007. 24704276

[53] Nakamura H, Lu M, Gwack Y, Souvlis J, Zeichner SL, Jung JU 2003 Global changes in Kaposi's sarcoma-associated virus gene expression patterns following expression of a tetracycline-inducible Rta transactivator. J Virol 77:4205–4220. doi:10.1128/JVI.77.7.4205-4220.2003. 12634378 PMC150665

[54] Brulois KF, Chang H, Lee AS, Ensser A, Wong LY, Toth Z, Lee SH, Lee HR, Myoung J, Ganem D, Oh TK, Kim JF, Gao SJ, Jung JU 2012 Construction and manipulation of a new Kaposi's sarcoma-associated herpesvirus bacterial artificial chromosome clone. J Virol 86:9708–9720. doi:10.1128/JVI.01019-12. 22740391 PMC3446615

[55] Sturzl M, Gaus D, Dirks WG, Ganem D, Jochmann R 2013 Kaposi's sarcoma-derived cell line SLK is not of endothelial origin, but is a contaminant from a known renal carcinoma cell line. Int J Cancer 132:1954–1958. doi:10.1002/ijc.27849. 22987579

[56] Myoung J, Ganem D 2011 Generation of a doxycycline-inducible KSHV producer cell line of endothelial origin: maintenance of tight latency with efficient reactivation upon induction. J Virol Methods 174:12–21. doi:10.1016/j.jviromet.2011.03.012. 21419799 PMC3095772

[57] Baquero-Perez B, Whitehouse A 2015 Hsp70 isoforms are essential for the formation of Kaposi's sarcoma-associated herpesvirus replication and transcription compartments. PLoS Pathog 11:e1005274. doi:10.1371/journal.ppat.1005274. 26587836 PMC4654589

[58] Vieira J, O'Hearn PM 2004 Use of the red fluorescent protein as a marker of Kaposi's sarcoma-associated herpesvirus lytic gene expression. Virology 325:225–240. doi:10.1016/j.virol.2004.03.049. 15246263

[59] Huang DW, Sherman BT, Lempicki RA 2009 Systematic and integrative analysis of large gene lists using DAVID bioinformatics resources. Nat Protoc 4:44–57. doi:10.1038/nprot.2008.211. 19131956

[60] Huang DW, Sherman BT, Zheng X, Yang J, Imamichi T, Stephens R, Lempicki RA 2009 Extracting biological meaning from large gene lists with DAVID. Curr Protoc Bioinformatics Chapter 13:Unit 13.11. doi:10.1002/0471250953.bi1311s27. 19728287

[61] Gould F, Harrison SM, Hewitt EW, Whitehouse A 2009 Kaposi's sarcoma-associated herpesvirus RTA promotes degradation of the Hey1 repressor protein through the ubiquitin proteasome pathway. J Virol 83:6727–6738. doi:10.1128/JVI.00351-09. 19369342 PMC2698570

[62] Hughes DJ, Wood JJ, Jackson BR, Baquero-Perez B, Whitehouse A 2015 NEDDylation is essential for Kaposi's sarcoma-associated herpesvirus latency and lytic reactivation and represents a novel anti-KSHV target. PLoS Pathog 11:e1004771. doi:10.1371/journal.ppat.1004771. 25794275 PMC4368050

[63] Nelson JD, Denisenko O, Bomsztyk K 2006 Protocol for the fast chromatin immunoprecipitation (ChIP) method. Nat Protoc 1:179–185. doi:10.1038/nprot.2006.27. 17406230

[64] Nelson JD, Denisenko O, Sova P, Bomsztyk K 2006 Fast chromatin immunoprecipitation assay. Nucleic Acids Res 34:e2. doi:10.1093/nar/gnj004. 16397291 PMC1325209

[65] Kumar P, Wood C 2013 Kaposi's sarcoma-associated herpesvirus transactivator Rta induces cell cycle arrest in G0/G1 phase by stabilizing and promoting nuclear localization of p27kip. J Virol 87:13226–13238. doi:10.1128/JVI.02540-13. 24067984 PMC3838229

[66] Hori T, Osaka F, Chiba T, Miyamoto C, Okabayashi K, Shimbara N, Kato S, Tanaka K 1999 Covalent modification of all members of human cullin family proteins by NEDD8. Oncogene 18:6829–6834. doi:10.1038/sj.onc.1203093. 10597293

[67] Bennett EJ, Rush J, Gygi SP, Harper JW 2010 Dynamics of cullin-RING ubiquitin ligase network revealed by systematic quantitative proteomics. Cell 143:951–965. doi:10.1016/j.cell.2010.11.017. 21145461 PMC3008586

[68] Soucy TA, Smith PG, Milhollen MA, Berger AJ, Gavin JM, Adhikari S, Brownell JE, Burke KE, Cardin DP, Critchley S, Cullis CA, Doucette A, Garnsey JJ, Gaulin JL, Gershman RE, Lublinsky AR, McDonald A, Mizutani H, Narayanan U, Olhava EJ, Peluso S, Rezaei M, Sintchak MD, Talreja T, Thomas MP, Traore T, Vyskocil S, Weatherhead GS, Yu J, Zhang J, Dick LR, Claiborne CF, Rolfe M, Bolen JB, Langston SP 2009 An inhibitor of NEDD8-activating enzyme as a new approach to treat cancer. Nature 458:732–736. doi:10.1038/nature07884. 19360080

[69] Blank JL, Liu XJ, Cosmopoulos K, Bouck DC, Garcia K, Bernard H, Tayber O, Hather G, Liu R, Narayanan U, Milhollen MA, Lightcap ES 2013 Novel DNA damage checkpoints mediating cell death induced by the NEDD8-activating enzyme inhibitor MLN4924. Cancer Res 73:225–234. doi:10.1158/0008-5472.CAN-12-1729. 23100467

[70] Ma T, Chen Y, Zhang F, Yang CY, Wang S, Yu X 2013 RNF111-dependent neddylation activates DNA damage-induced ubiquitination. Mol Cell 49:897–907. doi:10.1016/j.molcel.2013.01.006. 23394999 PMC3595365

[71] Hannah J, Zhou P 2009 Regulation of DNA damage response pathways by the cullin-RING ubiquitin ligases. DNA Repair (Amst) 8:536–543. doi:10.1016/j.dnarep.2009.01.011. 19231300 PMC2858918

[72] Le-Trilling VT, Megger DA, Katschinski B, Landsberg CD, Ruckborn MU, Tao S, Krawczyk A, Bayer W, Drexler I, Tenbusch M, Sitek B, Trilling M 2016 Broad and potent antiviral activity of the NAE inhibitor MLN4924. Sci Rep 6:19977. doi:10.1038/srep19977. 26829401 PMC4734293

[73] Grossmann C, Ganem D 2008 Effects of NFkappaB activation on KSHV latency and lytic reactivation are complex and context-dependent. Virology 375:94–102. doi:10.1016/j.virol.2007.12.044. 18321555 PMC2822626

[74] Lei X, Bai Z, Ye F, Xie J, Kim CG, Huang Y, Gao SJ 2010 Regulation of NF-kappaB inhibitor IkappaBalpha and viral replication by a KSHV microRNA. Nat Cell Biol 12:193–199. doi:10.1038/ncb2019. 20081837 PMC2815189

[75] Jin Y, He Z, Liang D, Zhang Q, Zhang H, Deng Q, Robertson ES, Lan K 2012 Carboxyl-terminal amino acids 1052 to 1082 of the latency-associated nuclear antigen (LANA) interact with RBP-Jkappa and are responsible for LANA-mediated RTA repression. J Virol 86:4956–4969. doi:10.1128/JVI.06788-11. 22379075 PMC3347342

[76] Kaul R, Verma SC, Robertson ES 2007 Protein complexes associated with the Kaposi's sarcoma-associated herpesvirus-encoded LANA. Virology 364:317–329. doi:10.1016/j.virol.2007.03.010. 17434559 PMC4067005

[77] Li Q, He M, Zhou F, Ye F, Gao SJ 2014 Activation of Kaposi's sarcoma-associated herpesvirus (KSHV) by inhibitors of class III histone deacetylases: identification of sirtuin 1 as a regulator of the KSHV life cycle. J Virol 88:6355–6367. doi:10.1128/JVI.00219-14. 24672028 PMC4093851

[78] Schmid M, Speiseder T, Dobner T, Gonzalez RA 2014 DNA virus replication compartments. J Virol 88:1404–1420. doi:10.1128/JVI.02046-13. 24257611 PMC3911613

[79] Krishnan HH, Naranatt PP, Smith MS, Zeng L, Bloomer C, Chandran B 2004 Concurrent expression of latent and a limited number of lytic genes with immune modulation and antiapoptotic function by Kaposi's sarcoma-associated herpesvirus early during infection of primary endothelial and fibroblast cells and subsequent decline of lytic gene expression. J Virol 78:3601–3620. doi:10.1128/JVI.78.7.3601-3620.2004. 15016882 PMC371072

[80] AuCoin DP, Colletti KS, Cei SA, Papouskova I, Tarrant M, Pari GS 2004 Amplification of the Kaposi's sarcoma-associated herpesvirus/human herpesvirus 8 lytic origin of DNA replication is dependent upon a cis-acting AT-rich region and an ORF50 response element and the trans-acting factors ORF50 (K-Rta) and K8 (K-bZIP). Virology 318:542–555. doi:10.1016/j.virol.2003.10.016. 14972523

